# HYPERTROPHIC LUPUS VULGARIS: AN UNUSUAL PRESENTATION

**DOI:** 10.4103/0019-5154.55644

**Published:** 2009

**Authors:** Vijay K Jain, Kamal Aggarwal, Sarika Jain, Sunita Singh

**Affiliations:** *From the Department of Dermatology, Venereology and Leprology, Pt. B.D. Sharma Post Graduate Institute of Medical Sciences, Rohtak, Haryana, India.*; 1 *From the Department of Pathology, Pt. B.D. Sharma Post Graduate Institute of Medical Sciences, Rohtak, Haryana, India.*

**Keywords:** *Cutaneous tuberculosis*, *hypertrophic*, *lupus vulgaris*

## Abstract

Lupus vulgaris is the most common form of cutaneous tuberculosis occurring in previously sensitized individuals with a high degree of tuberculin sensitivity. Various forms including plaque, ulcerative, hypertrophic, vegetative, papular, and nodular forms have been described. A 30-year-old male patient presented with a very large hypertrophic lupus vulgaris lesion over left side of chest since 22 years. Histopathological examination showed granulomatous infiltration without caseation necrosis. The Mantoux reaction was strongly positive. Hypertrophic lupus vulgaris of such a giant size and that too at an unusual site is extremely rare and hence is being reported.

## Introduction

Cutaneous tuberculosis forms a small proportion of extrapulmonary tuberculosis.[1] Studies from India report an incidence of cutaneous tuberculosis of 0.1%.[2] It takes different clinical forms depending on previous contact and the patient's immune status. It has been shown that lupus vulgaris is the most common form in adults.[2] It is a reinfection tuberculosis of the skin occurring in previously sensitized individuals with a high degree of tuberculin sensitivity and moderate immunity. A typical feature of lupus vulgaris is its extremely chronic course, characterized by slow but steady growth of the lesion over a period of years. Although plaque type is the common type, other variants such as vegetative, ulcerative, hypertrophic papular and nodular forms are also described. Hypertrophic lupus vulgaris has been reported only scarcely in the literature. We, herein report a case of a very large-sized hypertrophic lupus vulgaris present over the chest.

## Case Report

**Figure 1:**
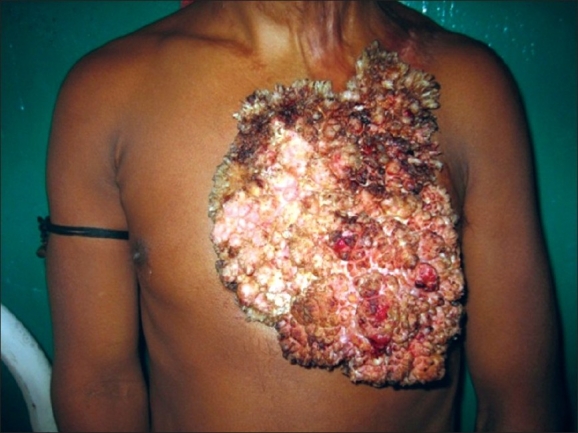
Clinical photograph showing the initial appearance of the large-sized growth on the left side of chest

**Figure 2:**
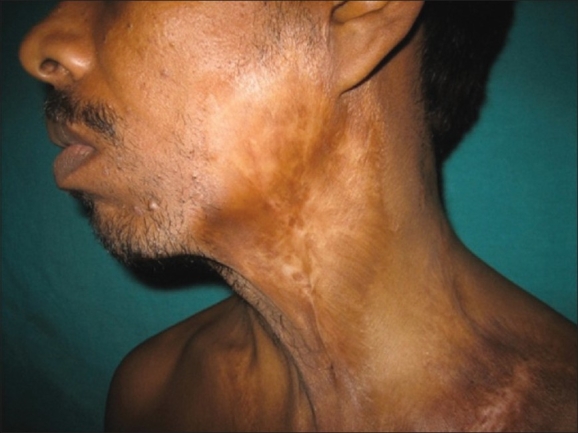
Atrophic scarring over left mandibular region and neck

A 30-year-old male, with no prior history of tuberculosis, presented with a growth of 22-year duration on the left side of the chest [Figure 1]. The lesion had steadily grown since it first appeared when he was 8-year-old. Similar, though smaller lesions were also present over bilateral mandibular regions, neck, and axilla, which on taking some treatment had resolved about 2 years back leaving behind atrophic scars [Figure 2]. The lesion was asymptomatic apart from discharge of foul smelling seropurulent material. There was no history of apparent trauma or previous history suggestive of tuberculous involvement of any other part of the body. The family history was not significant.

Dermatological examination revealed a solitary, 30 × 25 cm, verrucous, cauliflower-like growth over left side of the chest extending from just below the clavicle to the lower border of the thoracic cage. Surface showed multiple nodular infiltrations, which were covered with thick crusts. Removal of the crusts showed a friable tumor mass, soft in consistency and in the upper part showed multiple digitate projections, which were hard to palpate. Areas of atrophic scarring were present bilaterally symmetrically over mandibular region, neck, and axilla. There was no clinically significant lymphadenopathy. The general condition of the patient was good and no signs of systemic tuberculosis were demonstrated.

**Figure 3:**
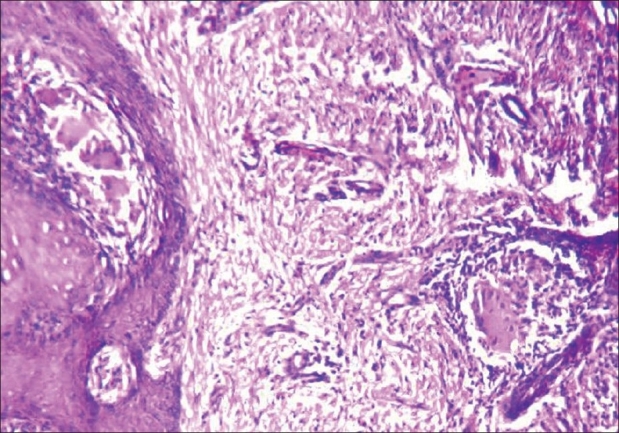
Skin biopsy showing epithelioid cell granulomas with Langhans giant cells in the upper dermis (H&E, ×400)

Hematological investigations showed anemia, raised erythrocyte sedimentation rate, positive C-reactive protein, and a normal chest X-ray; CECT thorax ruled out any infiltration into underlying musculature, lung fields or mediastinal structures. The Mantoux reaction was 19 × 17mm at 72h. Sputum for acid fast bacilli (AFB) was negative. A Ziehl–Neelsen's stained smear from the lesion was negative, as was also the culture on Lowenstein–Jensen medium after 6 weeks of inoculation. Superficial scrapings from the surface of the lesion were negative for fungus. ELISA for HIV was nonreactive. Histopathological examination of the biopsy specimen revealed changes characterized by epidermal acanthosis, hyperkeratosis, and papillomatosis. The dermis was marked by the presence of epithelioid cell granulomas with conspicuous Langhans type of giant cells, located primarily in the upper dermis. In addition, the dermis also showed infiltration by chronic inflammatory cells, mainly lymphocytes. No caseation necrosis was seen. Ziehl–Neelsen's stain did not reveal any AFB. No fungal cells were seen in the tissue sections [Figure 3].

**Figure 4:**
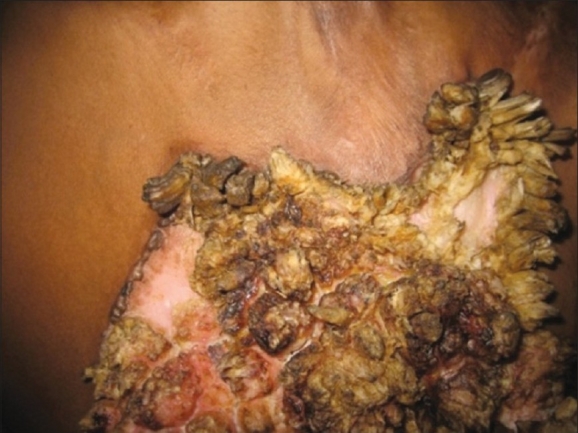
Clinical photograph after completion of ATT showing healing with atrophy

On the basis of the clinical, laboratory, and histopathological features, the diagnosis of hypertrophic lupus vulgaris was made. Patient was put on four-drug antituberculous therapy (ATT) for 6 months (owing to the large size of the lesion). After completion of therapy, lesion showed healing with atrophy at places [Figure 4].

## Discussion

Lupus vulgaris is a postprimary, paucibacillary form of skin tuberculosis arising in previously sensitized individuals with moderate immunity. Lesions arise by either contiguous extension of the disease from the underlying affected tissue or by hematogenous or lymphatic spread. In some instances, lupus appears over a primary inoculation site, but more than half of the cases follow other tuberculous diseases.[2] The face is the most commonly affected site in Western countries with a frequent affliction of the nose and cheeks. In India, however, lower extremities, especially buttocks are most frequently affected. It is characterized by macules or papules, with a brownish-red color and soft consistency that form larger plaques by peripheral enlargement and coalescence. Atrophic scarring, with or without ulceration, is a prominent feature of lupus vulgaris. It has diverse clinical variants such as plaque, hypertrophic (tumor-like), ulcerative, vegetative, papular, and nodular forms. The hypertrophic variety presents either as soft tumorous growths exhibiting a nodular knobby surface or showing epithelial hyperplasia with the production of hyperkeratotic masses.[2] The histopathology is characterized by the formation of tuberculoid granulomas composed of epithelioid cells and giant cells, usually of Langhans type. Caseation necrosis with in the tubercles is slight or absent. There is an associated infiltrate of lymphocytes. Secondary changes in the epidermis are common which becomes hyperplastic and shows acanthosis, hyperkeratosis, and papillomatosis. Tubercle bacilli are generally hard to demonstrate.[3] As lupus vulgaris is a paucibacillary form of cutaneous tuberculosis, demonstration of AFB on the smear prepared from the material from the lesion or culture on Lowenstein–Jensen medium seldom yields positive results. Therefore, microscopic pathology is the mainstay for diagnosis.

After a thorough search of literature, only few case reports of hypertrophic lupus vulgaris were found.[4–6] However, such a giant-sized lesion and that too at an unusual site is an extreme rarity.
